# Dosimetric evaluation of a novel high dose rate (HDR) intraluminal / interstitial brachytherapy applicator for gastrointestinal and bladder cancers

**DOI:** 10.1120/jacmp.v12i1.3360

**Published:** 2010-12-02

**Authors:** Sanaz Hariri Tabrizi, Seyyed Mahmoud Reza Aghamiri, Siamak Najarian, Ramin Jaberi

**Affiliations:** ^1^ Department of Radiation Medicine Engineering Shahid Beheshti University Tehran; ^2^ Faculty of Biomedical Engineering Amirkabir University of Technology Tehran; ^3^ Cancer Institute Medical University of Tehran Tehran Iran

**Keywords:** dosimetry, HDR brachytherapy applicator, well‐type chamber, radiochromic film

## Abstract

High dose rate (HDR) brachytherapy is one of the accepted treatment modalities in gastro‐intestinal tract and bladder carcinomas. Considering the shortcoming of contact brachytherapy routinely used in gastrointestinal tract in treatment of big tumors or invasive method of bladder treatment, an intraluminal applicator with the capability of insertion into the tumor depth seems to be useful. This study presents some dosimetric evaluations to introduce this applicator to the clinical use. The radiation attenuation characteristics of the applicator were evaluated by means of two dosimetric methods including well‐type chamber and radiochromic film. The proposed 110 cm long applicator has a flexible structure made of stainless steel for easy passage through lumens and a needle tip to drill into big tumors. The 2 mm diameter of the applicator is thick enough for source transition, while easy passage through any narrow lumen such as endoscope or cystoscope working channel is ensured. Well‐chamber results showed an acceptably low attenuation of this steel springy applicator. Performing absolute dosimetry resulted in a correlation coefficient of $R=0.9916(p‐\text{value}\approx 10^{-7})$ between standard interstitial applicator and the one proposed in this article. This study not only introduces a novel applicator with acceptable attenuation but also proves the response independency of the GAFCHROMIC EBT films to energy. By applying the dose response of the applicator in the treatment planning software, it can be used as a new intraluminal / interstitial applicator.

PACS number: 87.53.Bn, 87.53.Jw, 29.40.Cs

## I. INTRODUCTION

Surgery remains the cornerstone of successful treatment of gastrointestinal carcinomas. Achieving negative surgical resection margins is crucial.^(^ ^1^ ^)^ However, in case of locally advanced disease, local and locoregional recurrence rates cannot be frequently improved by more extensive surgery. Brachytherapy is known as an effective treatment modality for some cases of gastrointestinal cancers including: oesophagus,^(^ ^2^ ^–^ ^5^ ^)^ bile duct,^(^ ^6^ ^,^ ^7^ ^)^ rectum^(^ ^8^ ^–^ ^11^ ^)^ and anus cancers.^(^ ^10^ ^,^ ^12^ ^)^ Intraluminal brachytherapy is categorized under contact type method which can lead to insufficient tumor coverage in the case of large and invasive tumors.^(^ ^13^ ^,^ ^14^ ^)^ As an example, the high dose rate (HDR) brachytherapy of oesophagus is recommended for patients with advanced unresectable cancer^(^ ^2^ ^)^ who are indicated with tumor sizes deeper than 9 cm. Considering dose fall in this distance leads to an idea for inserting the applicator to the tumor depth, but without a need to perform surgery.

On the other hand, the candidates for interstitial brachytherapy of bladder who have solitary and histologically confirmed transitional cell carcinoma of ≤5 cm in diameter^(^ ^15^ ^)^, undergo an invasive method of applicator insertion. Thus for cases in which intraluminal brachytherapy may not cover the whole tumor volume, or as an alternative to invasive interstitial methods of brachytherapy, combining the advantages of each method appears to be effective. For all of the four brachytherapy capable sites recognized in gastrointestinal tract, use of endoscope for inserting the applicators is defined.^(^ ^7^ ^)^ As a result, an applicator was designed with the capability to travel a long distance through lumens of endoscope or cystoscope, while it also can be inserted into the predetermined depth of tumor. In this study, the dose distribution around this novel applicator was studied.

## II. MATERIALS AND METHODS

### A. The proposed applicator

**Figure 1:**
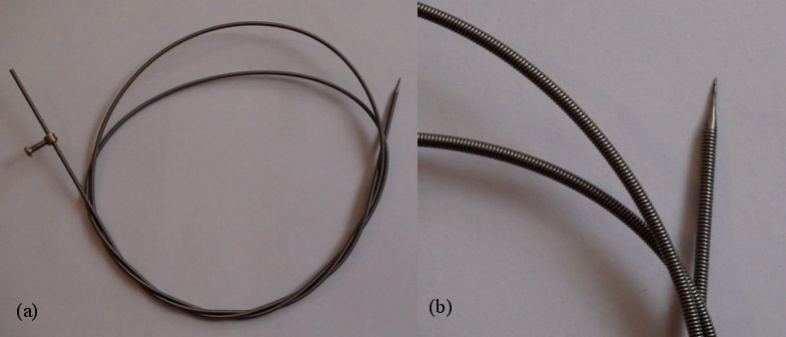
Novel applicator (a) with a springy structure, a needle tip and a stopper at the distal end for intraluminal / interstitial HDR brachytherapy; zoomed photo (b) from the tip of the applicator.

The introduced applicator (Fig. 1) can be used as both intraluminal and interstitial brachytherapy applicator. It is a 110 cm long, 2 mm diameter springy stainless steel hollow wire with a welded needle at the tip. Its length and diameter are suitable for passing through the working channel of endoscope or cystoscope probes. Also, the springy structure permits deep insertion of the applicator like a drill up to the predetermined depth which is settled by a stopper at the distal end.

Rectum application is another use, which can be mentioned. Analogous to the Nucletron colorectal applicator (Nucletron, Veenendaal, Netherlands),^(^ ^16^ ^)^ the one used in this study is flexible and thin enough to be used in a multiple channel array. The main difference between these applicators is their materials; while the Nucletron applicator is composed of silicone rubber, the proposed one is made from stainless steel to provide a mild stiffness. In addition, while swirling like a drill, using a needle at the tip makes it capable to be inserted into a desired depth in the tumor.

In fact, the wire of the proposed applicator is an ordinary accessory device in colonoscope packages. Therefore, the biocompatibility and availability were assured although no data were provided about its radiation attenuation characteristics. One of the main properties of an applicator is its radiation attenuation factor. Accordingly, dosimetric evaluation of the proposed applicator before clinical use was necessary. Two dosimetric evaluations were conducted: one relative to standard interstitial applicators by means of an HDR well‐type chamber and another quantitative dosimetry using radiochromic films.

### B. Well‐type chamber dosimetry

One means of measuring the relative strength of a radioactive source is to use a well‐type (reentrant) ionization chamber. Such chambers are commercially available and, when used with care, provide a very convenient means of assessing the strength of radioactive sources.^(^ ^17^ ^)^ Our applicator was connected via a connector to the channels of Flexitron HDR remote afterloader (Nucletron, Veenendaal, Netherlands). The relative dosimetry was performed using an HDR well‐type ionization chamber (model HDR1000 Plus, Standard Imaging, Middleton, WI). The chamber was calibrated at the ADCL laboratory before the measurements. Two Flexitron proprietary applicators including an interstitial and an oesophagus applicator were compared with the suggested one. Two readings were performed for each one while the ${\;}^{192}\text{Ir}$ source was positioned in approximately middle of the chamber. The dwell time of the source in each position was 20 seconds.

### C. Radiochromic film dosimetry

There were three sets of film pieces called experimental, calibration and background. Experimental films were irradiated in the experimental configuration to find out the resulted dose distribution by the applicator. The calibration films were pieces with the same dimension as the experimental ones irradiated by ${\;}^{192}\text{Ir}$ source itself or smaller ones irradiated by 6 and 18 MV photon beams. They were used to examine the absorbed dose as a function of film darkness or net optical density. Finally, there were two sets of background films with the same dimension as the corresponding irradiated ones (whether experimental or calibration), but these were not irradiated. The background film pieces were used in order to account for the environmental condition on the films and subtracting these effects from the net result of irradiation.

Absolute measurement was performed using GAFCHROMIC EBT film (International Specialty Products, Wayne, NJ, lot No. 37137‐021) and the ${\;}^{192}\text{Ir}$ source of HDR afterloader to obtain the dose distributions around the applicator. It was measured using film pieces between Plexiglas phantom slabs with varying thickness and, as a result, varying distance from the applicator surface. The phantom was $30\times 20\times 20\;{\text{cm}}^{3}$ to consider the full scatter effect. Handling the films was done based on the AAPM TG‐55 recommendations.^(^ ^18^ ^)^ Experimental films were cut in pieces of 12.6 cm ×1.3 cm about 24 hours prior to irradiation.^(^ ^19^ ^)^ The film orientation and alignment was noted to minimize the polarization effect.

The read‐out of the films was carried out with a time interval of at least 48 hours to ensure full color development.^(^ ^18^ ^,^ ^19^ ^)^ Analysis was performed using a Microtek 9800 XL flat‐bed document scanner (Microtek International Inc., Hsinchu City, Taiwan) based on the protocol described by Devic et al.^(^ ^20^ ^)^ and Lynch et al.^(^ ^21^ ^)^ Based on the proximity of film pieces to the applicator in the experiment, the region of interest (ROI) of each film was identified. In order to avoid large variance of pixel values, the ROI of nearer films to the source were smaller than the further.

The film images were analyzed using an in‐house MATLAB routine (The MathWorks, Natick, MA, MATLAB R2006b version 7.3). Since the absorbance spectra of GAFCHROMIC EBT peaks at 636 nm, the sensitivity is maximized by measurement with red light.^(^ ^18^ ^)^ The pixel values of the red channel were extracted and a 7×7 Wiener filter was applied for smoothing the background noise.^(^ ^20^ ^)^ The net optical density (NOD) was calculated based on Devic equations.^(^ ^20^ ^)^

#### C.1 Film calibration

Since the most accessible and reliable source for the calibration procedure was a linear accelerator (linac), we used linac photon beams with two different energies in addition to the HDR brachytherapy ${\;}^{192}\text{Ir}$ source. Because the film response is nearly energy independent from 6 MV photon down to 22 keV,^(^ ^22^ ^,^ ^23^ ^)^ the calibration curve for 6 MV photon can serve as a check on the calibration curve for ${\;}^{192}\text{Ir}$ energy. We have examined the 18 MV photon beam results as well to show a new independency range of EBT films to energy. Six film pieces were cut in $1\times 1\;{\text{cm}}^{2}$ dimensions and they were irradiated in a $10\times 10\;{\text{cm}}^{2}$ field of linac for each dose step of 0, 1, 1.5, 2, 3, 5, 7 and 10 Gy. The linac was calibrated by means of an ionization chamber prior to main experiment. The film pieces for both 6 and 18 MV energies were put in the maximum buildup region using a slab of solid phantom and the total phantom size was $30\times 30\times 20\;{\text{cm}}^{3}$. The pixel values for each calibration film were averaged over 31×31 adjacent pixels so that the distribution had a grid spacing of $5.27\times 5.27\;{\text{mm}}^{2}$. The net optical density (NOD) values were then calculated as described by Devic et al.^(^ ^20^ ^)^

In addition to linac photon beams, HDR brachytherapy ${\;}^{192}\text{Ir}$ source was used as a self‐calibrator for our experiment. The air kerma strength of the source was measured by an HDR well‐type ionization chamber prior to the main experiment, and it was used to validate the treatment planning software database. The film pieces in this procedure were cut identical to the experimental ones and they were positioned at the same distances from the applicator surface between solid water slabs. The sensitometric curve was drawn between 0.6 Gy and 7.3 Gy doses with nine steps. The obtained NOD standard deviation of each calibration film was within 0.91% for all doses.

#### C.2 Sensitometric curve

Three different methods to obtain the best‐suited sensitometric curve were examined. The three calibration curves characterizing the measured NOD as a function of dose were obtained based on the average of the six calibration films of linac and ${\;}^{192}\text{Ir}$ source calibration tests. GAFCHROMIC EBT shows a nonlinear relation between the net optical density and dose.^(^ ^24^ ^)^ There are several studies which fit a third order polynomial to the calibration data to convert NOD to dose.^(^ ^21^ ^,^ ^25^ ^,^ ^26^ ^)^ Using this method, an increasing incline was obtained in our experiment in higher doses, which is in contrast with the saturation effect in the films.

Another method to fit a curve to the delivered dose (D) versus measured netOD is using an analytical expression:^(^ ^27^ ^,^ ^28^ ^,^ ^20^ ^)^

(1) $$D_{\mathrm{fit}}=b.\mathrm{netOD}+c\cdot {\mathrm{netOD}}^{n}$$

where *b*, *c* and *n* are the fitting parameters. Since Devic et al.^(^ ^20^ ^)^ found that the fit procedure returns the best results for a fitting parameter of n=2.5, other two parameters were calculated. Because a correlation coefficient of less than 95% between measured points and fitting curve was achieved by this method, the third technique of drawing a sensitometric curve was examined.

**Figure 2:**
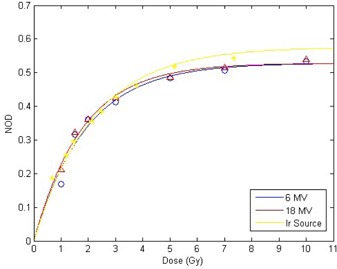
Sensitometric curves for 6 MV and 18 MV photon beams and ${\;}^{192}\text{Ir}$ source gamma ray with exponential fitting curve.

The coloration process of the EBT film crystals (i.e., the interaction of photons with the active component in EBT), is expected to follow Poisson statistics. Applying the single hit theory^(^ ^29^ ^,^ ^30^ ^)^ yields the next relation between OD and dose (D):

(2) $$\mathrm{OD}=\frac{c_{1}}{2(c_{2}/c_{1})}\left\{1-\text{exp}(-2(c_{2}/c_{1})D)\right\}$$

where $c_{1}$ is expressed in terms of ${\text{Gy}}^{-1}$ and $c_{2}$ in terms of ${\text{Gy}}^{-2}$. In accordance with the single hit theory,^(^ ^29^ ^)^ we can write Eq. (2) as:

(3) $$OD={\mathrm{OD}}_{\text{max}}\left\{1-\text{exp}(-c_{1}D/{\mathrm{OD}}_{\text{max}})\right\}$$

with the maximum optical density of ${\text{OD}}_{\max}=c_{1}/2(c_{2}/c_{1})$. The parameter ${\text{OD}}_{\text{max}}$ was initially intended to be a physical constant by definition,^(^ ^30^ ^)^ but here the parameter ${\text{OD}}_{\text{max}}$ will be a fit parameter.^(^ ^29^ ^)^ Applying the exponential equation to 6 and 18 MV data and also the data obtained by ${\;}^{192}\text{Ir}$ source calibration, resulted in Fig. 2. The mean ${\text{OD}}_{\text{max}}$ and $c_{1}$ parameters and their standard deviation as well as corresponding R‐square values were calculated by means of MATLAB software and they are summarized in Table 1.

**Table 1:** The ${\text{OD}}_{\text{max}}$ and $c_{1}$ parameters and the corresponding confidence bounds for 6 and 18 MV photon beams and ${\;}^{192}\text{Ir}$ gamma ray using exponential fitting.

*Energy*	$OD_{\max}$	*95% Confidence Bounds*	$c_{1}$ *(* ${\text{Gy}}^{-1}$ *)*	*95% Confidence Bounds*	$R^{2}$
6 MV	0.5285	0.4784 – 0.5787	0.2762	0.2173 – 0.335	96.36%
18 MV	0.5285	0.5031 – 0.5539	0.2981	0.2643 – 0.3319	98.78%
${\;}^{192}\text{Ir}(375\;\text{keV})$	0.5764	0.5442 – 0.6086	0.2672	0.2393 – 0.2952	98.12%

#### C.3 Dose distribution measurement around the applicator

The same arrangement as for the ${\;}^{192}\text{Ir}$ calibration was used for dose measurements around the proposed applicator. The films were placed horizontally between the slabs of solid phantom with predefined distances from the applicator surface and in the midline of it. The source was loaded to the final 10 cm of the applicator. On the day of the experiment, the source activity was 242.5 GBq. The dwell times were adjusted to deliver a dose of 2.5 Gy at 6 mm distance from the applicator surface. When the NOD values were calculated by Devic^(^ ^20^ ^)^ equations, the corresponding doses were determined by means of calibration curves. For conversion purpose, two situations were taken into account. The mean values for $c_{1}$ and ${\text{OD}}_{\text{max}}$ parameters were used in the inverse of Eq. (3). As another method to estimate the applicator performance, optimum values for ${\text{OD}}_{\text{max}}$ and $c_{1}$ parameters were determined based on finding the least deviation from the corresponding standard applicator dose distribution which was acquired from the treatment planning software output. These values were obtained within 95% confidence limits of the two parameters.

## III. RESULTS

### A. Well‐type chamber dosimetry

The readings from three applicators were compared. The results are summarized in Table 2. The oesophageal applicator is made of plastic, while the standard interstitial is a rigid steel rod. As a result, the suggested applicator which is a flexible springy steel wire showed a reading between those two. Therefore, we found out that the suggested applicator shows an acceptably low attenuation factor. The following radiochromic film experiment confirmed this result.

**Table 2:** Comparison of three applicators including two standard interstitial and intraluminal applicators and the suggested applicator based on the well‐chamber readings.

*Applicators*	*Readings (nC)*	*Standard Deviation (nC)*
Oesophageal	19.61	0.01
Interstitial	17.97	0.01
Suggested	18.61	0.2

### B. Radiochromic film dosimetry

**Figure 3:**
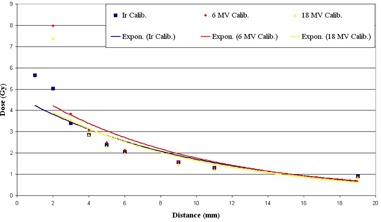
Comparison between measured dose from the proposed applicator using three calibration methods and mean value fitting parameters.

All the films, including the experimental and calibration, were scanned in both horizontal and vertical directions. As the pixel values in vertical position were less than in horizontal direction and according to GAFCHROMIC EBT film website^(^ ^31^ ^)^ recommendation, the horizontal values were taken into account. As discussed before, two situations were considered for comparison of standard interstitial applicator and our intraluminal / interstitial one. Using the mean values for fitting parameters in Eq. (3), Fig. 3 was obtained. It shows the measured dose transmitted through the proposed applicator calculated by three different methods of photon beams and ${\;}^{192}\text{Ir}$ source calibration. It is apparent that, except in the near vicinity to the applicator surface (i.e., 2 mm distance), there is a very good agreement between the results from these three methods of calibration. Not considering the distinct value, the percent of difference between ${\;}^{192}\text{Ir}$ source calibration and 6 and 18 MV photon beams is 3.08% and 4.77%, respectively.

As another approach, based on the least absolute difference between the calculated dose from inversion of the Eq. (3) and the Flexiplan program for standard applicator, optimum values for ${\text{OD}}_{\text{max}}$ and $c_{1}$ parameters were obtained. These values are summarized in Table 3.

**Table 3:** Optimum values in sensitometric equation for 6 MV and 18 MV photon beams and ${\;}^{192}\text{Ir}$ source calibration methods.

*Calibration Method*	$OD_{\max}$	$C_{1}$
6 MV	0.5495	0.2684
18 MV	0.5482	0.2743
${\;}^{192}\text{Ir}$ source	0.5495	0.2684

**Figure 4:**
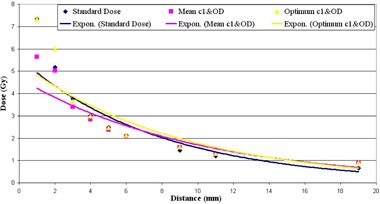
Comparison of standard interstitial and proposed applicator while the mean and optimum values for calibration fitting curves were used.

Figure 3 shows a good agreement between results from three calibration methods; hereafter, the ${\;}^{192}\text{Ir}$ curve was chosen for the proposed applicator evaluation. Figure 4 shows the flexible standard interstitial applicator dose distribution (with “Standard dose” legend) in comparison with the measured results from our applicator. It shows the comparison considering both situations of taking the mean fitting parameters in Eq. (3) into account (with “Mean $c_{1}$ & OD” legend) and using the calculated optimum ones (with “Optimum $c_{1}$ & OD” legend). All the data points in Figs. 3 and 4 are fitted by an exponential equation. This fitting curve relates to the exponential decreasing nature of the electromagnetic radiations by increasing the thickness of an absorber they pass through.

Using the best values for variable parameters in Eq. (3), the difference between standard and suggested applicator is negligible (R=0.9916). But this difference increases, although is still acceptable (R=0.9864), using the mean values for ${\text{OD}}_{\text{max}}$ and $c_{1}$ parameters. In other words, the percent of difference between standard dose and the obtained dose from our applicator in the case of using optimum calibration parameters is 8.83%, while it is 11.40% for the mean value parameters. The only significant disagreement between the data points is seen in the vicinity of the applicator in about 1 mm distance from its surface.

## IV. DISCUSSION

A novel two‐purpose HDR brachytherapy applicator was proposed with the application in gastrointestinal tract or bladder or any luminal part of the body. The flexible and springy structure and the use of a needle at the applicator tip allow conformal HDR brachytherapy treatment of deep‐seated and big tumors. Because the main issue about brachytherapy applicators is determining the attenuation factor, the dosimetric properties of this intraluminal / interstitial HDR brachytherapy applicator have been investigated. The dosimetric characteristics were examined using well‐type chamber and GAFCHROMIC EBT film. The well‐type chamber results show that this applicator has an acceptably low attenuation in comparison with two other standard applicators. This means that by complete evaluation of dose distribution around the applicator and defining it for the treatment planning system software, one can use it as a new HDR applicator.

As a complement, film dosimetry was done in the range 0–19 mm from the applicator surface. A similar response of the applicator to a proprietary interstitial applicator confirmed our assertion about acceptable dose response of the proposed applicator. Different slope of dose reduction of the two applicators relates to their different material composition, which is evident for both parameter sets (Fig. 4). While the suggested applicator is from stainless steel, the standard one is from plastic. This causes a different linear attenuation coefficient for each applicator. As a result, predominance of radiation attenuation in near distances from the applicator surface leads to dose reduction in the suggested applicator, while beam hardening characteristic causes increased absorbed dose in farther distances. This condition is more pronounced when mean values for fitting parameters are used in dose calculation. Since there is a high‐dose gradient around the ${\;}^{192}\text{Ir}$ source, a discrepancy is seen about 2 mm from the applicator surface between measured and standard doses. The problem can be reduced by using more data points in the low‐dose part of the calibration curve.

## V. CONCLUSIONS

We obtained good agreement between the standard interstitial applicator and the one in this study. Introducing an intraluminal applicator with the capability to use as an interstitial one opens up a new perspective in brachytherapy field. None of the available applicators can be inserted into the tumor depth while it travels a long distance from the origin. Although the applicator shows an acceptable dosimetric characterization, clinical trials are inevitable to examine the efficacy of using it in brachytherapy treatments. In addition to the main purpose of this paper, we have shown energy independent response of the GAFCHROMIC EBT film as another confirmation to its manufacturer's claim but with a wider energy range.

## ACKNOWLEDGMENTS

The authors would like to thank Dr. Mehrdad Azmi, the digestion specialist (Cancer Institute of Tehran University) and Dr. Hamid Reza Dehghan, the radiation oncologist (Medical University of Iran) who were reliable medical consultants. Also the kind assistance of Mr. Siamak Hajizadeh, Ms. Fatemeh Ghahremani and Mr. Seyed Abolfazl Saneii are appreciated.
